# A Very-low-energy Fast Involves Increased Adipose Inflammatory Gene Expression: A 6-day Feeding Trial (FASTOMICS-6)

**DOI:** 10.1210/clinem/dgaf570

**Published:** 2025-10-28

**Authors:** Marianne Bråtveit, Pouda P Strømland, Johnny Laupsa-Borge, Lillian Skumsnes, Vigdis H Dagsland, Silje Kvistad, Elinor C Vogt, Adrian McCann, Håvard L Thorsen, Andreas P Diamantopoulos, Bjørn Gunnar Nedrebø, Gunnar Mellgren, Simon N Dankel

**Affiliations:** Centre for Nutrition, Department of Clinical Science, University of Bergen, 5021 Bergen, Norway; Hormone Laboratory, Department of Medical Biochemistry and Pharmacology, Haukeland University Hospital, 5021 Bergen, Norway; Hormone Laboratory, Department of Medical Biochemistry and Pharmacology, Haukeland University Hospital, 5021 Bergen, Norway; Centre for Nutrition, Department of Clinical Science, University of Bergen, 5021 Bergen, Norway; Bevital AS, 5068 Bergen, Norway; Department of Research and Innovation, Haugesund Hospital, 5528 Haugesund, Norway; Department of Gastrointestinal Surgery, Haugesund Hospital, 5528 Haugesund, Norway; Deparment of Immunology and Transfusion Medicine, Haukeland University Hospital, 5021 Bergen, Norway; Department of Medicine, Haukeland University Hospital, 5021 Bergen, Norway; Bevital AS, 5068 Bergen, Norway; Department of Gastrointestinal Surgery, Haugesund Hospital, 5528 Haugesund, Norway; Department of Infectious Diseases, Akershus University Hospital, 1478 Lørenskog, Norway; Department of Medicine, Haugesund Hospital, 5528 Haugesund, Norway; Centre for Nutrition, Department of Clinical Science, University of Bergen, 5021 Bergen, Norway; Hormone Laboratory, Department of Medical Biochemistry and Pharmacology, Haukeland University Hospital, 5021 Bergen, Norway; Centre for Nutrition, Department of Clinical Science, University of Bergen, 5021 Bergen, Norway; Hormone Laboratory, Department of Medical Biochemistry and Pharmacology, Haukeland University Hospital, 5021 Bergen, Norway

**Keywords:** fasting, calorie restriction, adipose tissue, gene expression, metabolomics, obesity

## Abstract

**Background:**

Fasting and ketogenic diets may have health benefits, but we lack a deeper understanding of tissue mechanisms and associated metabolome changes, particularly in humans.

**Objective:**

Determine changes in subcutaneous adipose tissue (SAT) gene expression, plasma metabolomics, and circulating serum markers after a 6-day very-low-energy fast.

**Methods:**

13 patients with obesity (mean body mass index = 41.6) underwent a week-long in-hospital feeding study (600 kcal/day). Body composition was assessed by bioimpedance. Blood and SAT were collected on days 1 and 7 for metabolomics (gas chromatography coupled with tandem mass spectrometry) and transcriptomics (RNA-sequencing). Biological pathways were inferred from affected gene sets (gene set enrichment analysis) and gene networks that correlated with plasma metabolites.

**Results:**

Relative mean change (95% CI) in fat mass and fat-free mass was -2.07% (-2.93 to -1.20) and -4.42% (-5.53 to -3.28), respectively. Marked significant changes (relative mean change [95% CI]) were observed in total ketone bodies (acetoacetate + β-hydroxybutyrate) (+615% [366-999]), fasting insulin (-41.2% [-53.1 to -26.4]) and homeostatic model assessment for insulin resistance (-31.8% [-41.4 to -20.6]), as well as α-hydroxybutyrate (+103% [69.9-142]) and the advanced glycation end-products carboxyethyl-lysine (-46.0% [-53.6 to -37.2]) and carboxymethyl-lysine (-50.9% [-59.8 to -40.1]). Gene set enrichment analysis indicated upregulated inflammatory responses, and downregulated oxidative phosphorylation, adipogenesis, fatty acid metabolism, and mTORC1 signaling (*P* < .01) in SAT.

**Conclusion:**

After a 6-day very-low-energy fast, adipose inflammatory gene expression increased concomitant with decreased plasma advanced glycation end-products and improved insulin sensitivity. These data suggest a role for inflammatory and autophagy-associated pathways in the metabolic adaptation to a ketogenic very-low-energy fast.

**Clinical Trial Registry number:**

NCT02671279 (https://clinicaltrials.gov/study/NCT02671279)

Fasting, which can encompass a very-low-energy diet, alternate-day fasting, intermittent fasting, and other forms of periodic caloric desistance, has gained popularity in recent years amid the global surge in obesity and metabolic diseases. Fasting induces a metabolic shift from using liver-derived glucose to the breakdown of triglycerides in adipose tissue (AT), releasing free fatty acids and glycerol that are used for energy production in low-glucose conditions (1). Fasting leads to weight loss (2) and has been associated with several health benefits, including improved lipid profile (2), increased insulin sensitivity (2), and a delay in hallmarks of aging (3). Rodent studies suggest that these effects are at least partly independent of weight loss per se (4, 5), but the mechanisms are yet to be elucidated, particularly in humans.

During different phases of fasting or very-low-energy intake, the body's physiological response includes a decrease in both postprandial and fasting insulin concentrations, an increase in counterregulatory hormones like glucagon, a progressive increase in ketogenesis, and a gradual increase in gluconeogenesis. This includes breakdown of liver glycogen to glucose as well as muscle protein to provide substrates for gluconeogenesis (1). These profound switches in energy substrate production involve an orchestrated response across tissues and between metabolic and immune cells (6) and changes in a wide range of tissue-derived proteins in the circulation (7). Some studies, as highlighted in a recent scoping review (8), have also suggested a transient increase in systemic inflammation, but there are limited data on such changes on the tissue level. As demonstrated mostly in animal models, the responses to fasting also involve the breakdown and recycling of cellular components by lysosomes within the cell, referred to as autophagy (9), which, during times of nutrient deprivation or cell stress, contributes with nutrient provision and elimination of cellular waste products to secure cell survival (10). By clearing out cellular waste and promoting repair, autophagy is believed to protect against age-related diseases and promote cellular health because age-related reduction in autophagic flux may contribute to the accumulation of protein aggregates such as advanced glycation end-products (AGEs), and dysfunctional organelles (11).

AT plays a critical role in whole-body energy homeostasis (12). Yet, few studies have explored changes in human AT function in response to fasting, and it remains uncertain whether fasting activates processes such as autophagy in human AT (13). Controlled fasting intervention studies that include tissue biopsies as well as circulating biochemical variables, coupled with a multiomics approach, can provide new comprehensive insights into the physiological responses to fasting. The objective of this in-hospital controlled low-energy feeding study was therefore to determine transcriptomic changes in AT following a 6-day very-low-energy fasting intervention, and associated changes in plasma metabolites including amino acids, tricarboxylic acid (TCA)-cycle metabolites and AGEs.

## Materials and Methods

### Study Population and Intervention

This study was an in-hospital feeding trial conducted from August 2016 to August 2018 in accordance with the Declaration of Helsinki and was approved by the Regional Ethics Committee in Western Norway (2015/361 Rec West). The study protocol was registered at ClinicalTrials.gov (NCT02671279). Written informed consent was collected from all participants before enrollment. Inclusion criteria were males and females (defined by biological characteristics) aged 18 to 60 years with severe obesity (body mass index = 40-46) and fasting glucose <7 mmol/L. Exclusion criteria were known type 2 diabetes, treatment with antidiabetic medication, and allergy to substances in the planned study diet. Patients included in the study consumed their habitual diet and were assumed to remain weight stable until the intervention. The intervention consisted of a fasting-mimicking diet of 400 to 600 kcal/day (44 g of protein/day, 54 g carbohydrate/day, and 23 g fat/day), where all food was provided for 6 full days. Patients were served 5 protein-based meals per day, primarily with high-protein liquid products such as high-protein yogurt and soups. To support hydration, participants were instructed to drink water regularly between meals. Participants were scheduled for bariatric surgery after completion of the intervention and moved freely within the hospital at a low to moderate activity level.

### Anthropometry

Height was measured at baseline in upright position to the nearest centimeter using a wall stadiometer (SECA, Hamburg, Germany). Body weight, including measurements of fat-free mass and fat mass, was monitored daily from baseline to day 7 using bioelectrical impedance (Tanita, Body Composition Analyzer BC-418).

### Biochemical Analyses

Blood samples were taken on the day of admission and at day 7 after completing 6 full days of the dietary intervention. The samples were collected in the morning after an overnight fast (minimum 8 hours), and participants were instructed not to eat or drink anything except water after 10 Pm the previous day. Blood samples were stored at −80 °C until analyzed. Routine serum biochemical analyses were conducted according to standardized procedures at the Department for Medical Biochemistry and Pharmacology, Haukeland University Hospital, Bergen, Norway, whereas study-specific analyses of plasma metabolites including ketone bodies, amino acids, carboxylic acids, TCA-cycle intermediates, and AGEs were performed by Bevital AS, Bergen, Norway (www.bevital.no). All plasma metabolites were quantified using gas chromatography coupled with tandem mass spectrometry using a previously published method (14). Calculation of homeostatic model assessment for insulin resistance (HOMA2-IR), homeostatic model assessment of insulin sensitivity (HOMA2-%S), and homeostatic model assessment of β-cell function (HOMA2-%B) was done using the HOMA Calculator v2.2.3 (http://www.dtu.ox.ac.uk/homacalculator/index.php).

### Adipose Tissue Biopsies, RNA Extraction, and Bulk RNA-sequencing

Biopsies of superficial subcutaneous AT (SAT) (2-3 g) were taken by surgical excision under local anesthesia (xylocaine 10 mg/mL with adrenalin), from the same area but on opposite sides of the abdomen on the day of admission and at day 7. The tissues were immediately snap-frozen and stored at −80 °C. The total RNA content of the frozen AT biopsies was purified using the RNeasy Mini kit (Qiagen, 74116) with additional on-column DNase-I treatment (Qiagen, 79 254) at 25 °C to remove genomic DNA. RNA purity and integrity were assessed using an RNA 6000 Nano kit (Agilent Technologies, 5067-1511) on the 4200 TapeStation (Agilent, Santa Clara, CA, USA). The cDNA libraries were prepared using the Illumina Stranded mRNA Ligation kit with 350 ng of input RNA. All samples were quality controlled by the 4200 TapeStation using the DNA ScreenTape Analysis. The final cDNA libraries were additionally quantified with the KAPA Library Quantification Kit (Roche, USA) for Illumina sequencing platforms. All samples were paired-end sequenced (2 × 100 bp) on the Illumina NovaSeq 6000 system (Illumina, San Diego, CA, USA). Run data from NovaSeq 6000 were demultiplexed by Illumina bcl2fastq software, and the Fastq files were quality controlled by FastQC and MultiQC. Reads were aligned to GRCh38 and the human GENCODE v. 26 reference genome and transcriptome using HISAT2 2.2.1, before submission to subread v.1.5.2 for feature counts calculation, resulting in a raw read count matrix.

### Statistical Methods

The primary outcomes reported in this study are changes in SAT gene expression and secondary outcomes are relative changes in plasma metabolomics and other circulating serum markers after a 6-day very-low-energy fast. Data on continuous variables are presented as geometric means (1 SD ranges), arithmetic means (SD), and relative changes (95% CIs) from baseline to day 7. The geometric SD ranges were calculated by dividing and multiplying the geometric means with the geometric SD factors to obtain the lower and upper limits, respectively (15). Sex was defined by the sex assigned at birth and presented as counts. All statistical analyses were conducted with R version 4.2.2 (https://www.r-project.org/), and data transformation and exploration were done by using the *tidyverse* packages (https://tidyverse.tidyverse.org). Change scores from baseline to follow-up in absolute and relative terms for serum biochemical variables and plasma metabolites were analyzed by linear regression using the *gls function* in the *nlme* package (v. 3.1-160). The model included “time” as the main term of interest and was adjusted for age and sex. Because most biochemical variables fit a log-normal (multiplicative) distribution equally well or better than a normal (additive) distribution, values were transformed by the natural logarithm before the analyses of relative change scores (15). In the tables and main text, we report changes from baseline to 7 days as percentages calculated from the regression coefficients (ie, the average of log-ratios) by the formula 100×(exp^estimate^ −1). In the figures, the results are shown as sympercents (s%), which are additive and symmetric percentage changes calculated as the difference between the natural logs of 2 numbers multiplied by 100 (100 × ln(a)—100 × ln(b)), making it straightforward to present and interpret without back transformation (16). A *P* value <.05 was considered significant. To explore whether changes in significantly altered plasma metabolites correlated with changes in other serum biochemical variables, Spearman correlation analyses of the s% change in these plasma metabolites against the s% change in serum biochemical variables were performed.

The raw read count matrix derived from the RNA-sequencing was submitted to the DESeq2 (v. 1.38.3) (17), normalized, and filtered by the gene expression of at least 10 read counts for each sample and the sum value of ≥ 6 through each gene for all samples. The identified genes were then ranked based on the log2fold value for gene set enrichment analysis (GSEA), which was performed using the *clusterprofiler* (18) package (v. 4.6.2) based on predefined Hallmark gene sets at MSigDB (19) with a *P* value cutoff <.05 and a minimum gene set size of 25. Raw *P* values were adjusted for multiplicity by controlling the false discovery rate (FDR) with the Benjamini-Hochberg method. The complete gene list was also subjected to weighted gene coexpression network analysis (WGCNA) using the *WGCNA* package (v.1.73) to identify gene coexpression network modules, which could subsequently be correlated with plasma ketones and with the most significantly altered metabolites in a module-trait analysis. For the module-trait relationships showing a Spearman correlation coefficient of *β* > .55 or *β* < -.55, a pathway overrepresentation analysis of the genes in each module was performed, based on the same predefined Hallmark gene set. The overrepresentation analysis was done using the *enrichr* function from the *clusterprofiler* package with Benjamini-Hochberg correction and a *P* value cutoff of <.05.

The figures were made in R using the packages *ggplot2* v.3.51, *WGCNA,* and *clusterprofiler* and in Illustrator v.29.2.1. Vector graphics was created with BioRender.com.

## Results

### Patient Characteristics

A total of 15 patients were included and completed the study, of which 2 patients were excluded from the analyses because of missing plasma metabolite and RNA-sequencing data, leaving 13 patients (4 males and 9 females) for the metabolite analyses. Twelve of these 13 patients (3 males and 9 females) were included in the gene expression analyses; 1 patient had SAT samples of low RNA quality. A flow chart demonstrating the selection of data included in the analyses is found in Fig. S1 (20).

**Figure 1.**
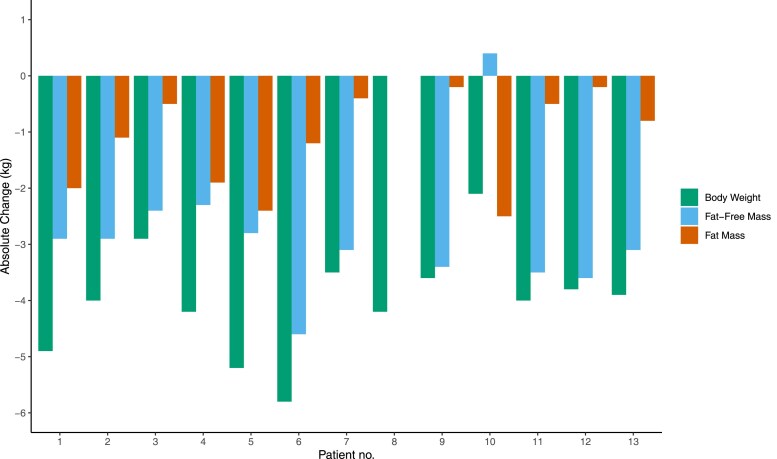
Bar plot showing the individual absolute changes in body weight, fat mass, and fat-free mass in kilograms throughout the intervention. The first bar per patient number (green color) refers to change in body weight, the second bar (blue color) per patient number refers to change in fat-free mass, and the third bar per patient (orange color) refers to change in fat mass.

The included patients were between 30 and 54 years (mean ± SD 45 ± 7.0) and had a mean (SD) body weight of 120 (14.1) kg. Baseline characteristics for all participants and relative change scores from baseline to day 7 are presented in Table 1. Total fat mass and fat-free mass were significantly reduced (relative mean change [95% CI]) by -2.07% [-2.93 to -1.20] and -4.42% [-5.53 to -3.28], respectively (Table 1), with a similar and significant total body weight reduction across participants (Fig. 1). Notably, except for 1 participant who gained a small amount of fat-free mass during the intervention, the loss of fat-free mass was largely similar across participants (range: -2.3 to -4.6 kg) (Fig. 1), whereas loss of fat mass varied from -0.2 kg to -2.5 kg (Fig. 1).

**Table 1.** Relative changes in anthropometrics and circulating biochemical variables^a^ after six full days of fasting

Variable	Baseline, gMean (1 SD ranges)	Baseline, mean (SD)	Day 7, gMean (1 SD ranges)	Day 7, mean (SD)	Relative change*^b^*	*P* value*^c^*
BMI, kg/m*^b^*	41.6 (39.9, 43.3)	41.6 (1.69)	40.2 (38.6, 41.8)	40.2 (1.64)	-3.33 (-3.65 to -3.00)	<.001
Body weight, kg	119 (106, 134)	120 (14.1)	115 (103, 129)	116 (13.4)	-3.33 (-3.65 to -3.00)	<.001
Fat mass, kg	55.0 (47.1, 64.3)	55.6 (8.28)	54.1 (45.9, 63.7)	54.8 (8.55)	-2.07 (-2.93 to -1.20)	<.001
Fat-free mass, kg	63.6 (54.3, 74.5)	64.3 (10.5)	61.3 (51.9, 72.3)	62.0 (10.7)	-4.42 (-5.53 to -3.28)	<.001
Acetoacetate, µmol/L	43.5 (23.0, 82.3)	52.7 (35.6)	225 (111, 458)	272 (150)	419 (259-649)	<.001
β-hydroxybutyrate, µmol/L	64.4 (31.2, 133)	82.7 (65.0)	550 (237, 1276)	712 (462)	755 (431-1275)	<.001
Total ketones, µmol/L*^e^*	109 (55.6, 215)	135 (96.3)	781 (354, 1724)	984 (605)	615 (366-999)	<.001
CRP, mg/L	6.96 (4.65, 10.4)	7.46 (2.70)	8.01 (4.47, 14.4)	9.15 (4.38)	15.1 (-3.51 to 37.3)	.132
Albumin, g/L	43.4 (41.7-45.1)	43.4 (1.66)	44.8 (43.1, 46.6)	44.9 (1.77)	3.37 (2.03-4.71)	<.001
Creatinine, µmol/L	72.8 (65.0-81.4)	73.2 (7.89)	75.1 (64.8, 87.0)	75.9 (11.4)	3.22 (-1.85 to 8.55)	.231
Glucose, mmol/L	6.23 (5.54-7.01)	6.27 (0.72)	5.29 (4.77-5.86)	5.32 (0.55)	-15.1 (-19.9 to -10.1)	<.001
Insulin, mIU/L	23.2 (15.0-35.8)	25.0 (9.05)	13.6 (7.17-25.8)	15.9 (8.32)	-41.2 (-53.1 to -26.4)	<.001
Insulin C-peptide, nmol/L	1.40 (1.00-1.98)	1.48 (0.46)	1.01 (0.65-1.56)	1.10 (0.48)	-28.2 (-37.5 to -17.5)	<.001
HOMA2-IR	3.30 (2.29-4.75)	3.49 (1.16)	2.25 (1.43-3.55)	2.48 (1.16)	-31.8 (-41.4 to -20.6)	<.001
HOMA2-%S	30.3 (21.0-43.6)	32.4 (13.6)	44.4 (28.2-70.0)	48.9 (23.2)	46.5 (25.8-70.7)	<.001
HOMA2%B	140 (113-174)	143 (26.9)	149 (114-195)	154 (36.8)	6.35 (-1.12 to 14.4)	.112
GT, U/L	29.7 (17.1-51.5)	34.1 (19.4)	28.4 (17.5-46.0)	31.4 (13.9)	-4.29 (-12.2 to 4.31)	.329
Cholesterol, mmol/L	5.20 (4.40-6.15)	5.27 (0.92)	4.99 (4.15-6.01)	5.07 (0.91)	4.01 (-9.88 to 2.24)	.217
HDL, mmol/L	1.10 (0.93-1.28)	1.11 (0.17)	0.96 (0.81-1.15)	0.98 (0.17)	-12.1 (-16.8 to -7.10)	<.001
LDL, mmol/L	3.40 (2.69-4.30)	3.49 (0.89)	3.30 (2.58-4.23)	3.39 (0.81)	-2.92 (-11.2 to 6.12)	.521
TAG, mmol/L	1.75 (1.19-2.57)	1.89 (0.88)	1.36 (0.98-1.89)	1.44 (0.50)	-22.2 (-33.4 to -9.04)	.005
TAG/HDL-ratio, mmol/L	3.65 (2.35-5.68)	4.06 (2.39)	3.23 (2.12-4.93)	3.54 (1.81)	-11.5 (-25.7 to 5.39)	.184
TSH, mIU/L	1.43 (0.98-2.08)	1.52 (0.52)	1.41 (1.03-1.93)	1.47 (0.42)	-1.67 (-10.8 to 8.40)	.738
FT4, pmol/L	14.9 (12.6-17.6)	15.1 (2.58)	17.0 (14.6-19.8)	17.2 (2.69)	14.6 (10.5-18.8)	<.001

Abbreviations: CRP, C-reactive protein; FT4, free thyroxine; GT, glutamyl transferase; HDL, high-density lipoprotein; HOMA2-%B, homeostatic model assessment of β-cell function; HOMA2-%S, homeostatic model assessment of insulin sensitivity; HOMA2-IR, homeostatic model assessment for insulin resistance; LDL, low-density lipoprotein; TAG, triacylglycerol.

^*a*^The biochemical variables were measured in serum, except the ketone bodies acetoacetate and β-hydroxybutyrate, which were measured in plasma. All data were analyzed with linear regression adjusted for age and sex. Values were transformed by the natural logarithm before the analyses.

^*b*^Relative model-adjusted mean change scores (95% CIs) from baseline to follow-up as percentages calculated from the model estimates: % = (exp^estimate—^1)×100.

^*c*^ *P* values for adjusted relative changes from baseline to day 7.

^d^Unadjusted aritmethric means (SDs) and absolute changes (95% CIs) of untransformed data as well as unadjusted gMean and relative changes (95% CIs) from the linear regression models are found in Tables S1-3 (20).

^*e*^Total ketones means acetoacetate plus β-hydroxybutyrate.

For biochemical variables, a statistically significant increase was observed after 6 full days of fasting for albumin, HOMA2-%S, and free T4 (FT4), whereas a significant reduction was observed for glucose, insulin, insulin C-peptide, HOMA2-IR, triacyclglycerols, and high-density lipoprotein cholesterol (all *P* < .005). We also observed significant increases in the ketone bodies acetoacetate and β-hydroxybutyrate (β-HB), indicating fasting-induced ketosis by day 7 (*P* < .001) (Table 1). Unadjusted absolute and relative change scores, as well as absolute adjusted change scores from baseline to day 7 are found in Tables S1-S3 (20).

### Gene Expression Analysis

**Figure 2.**
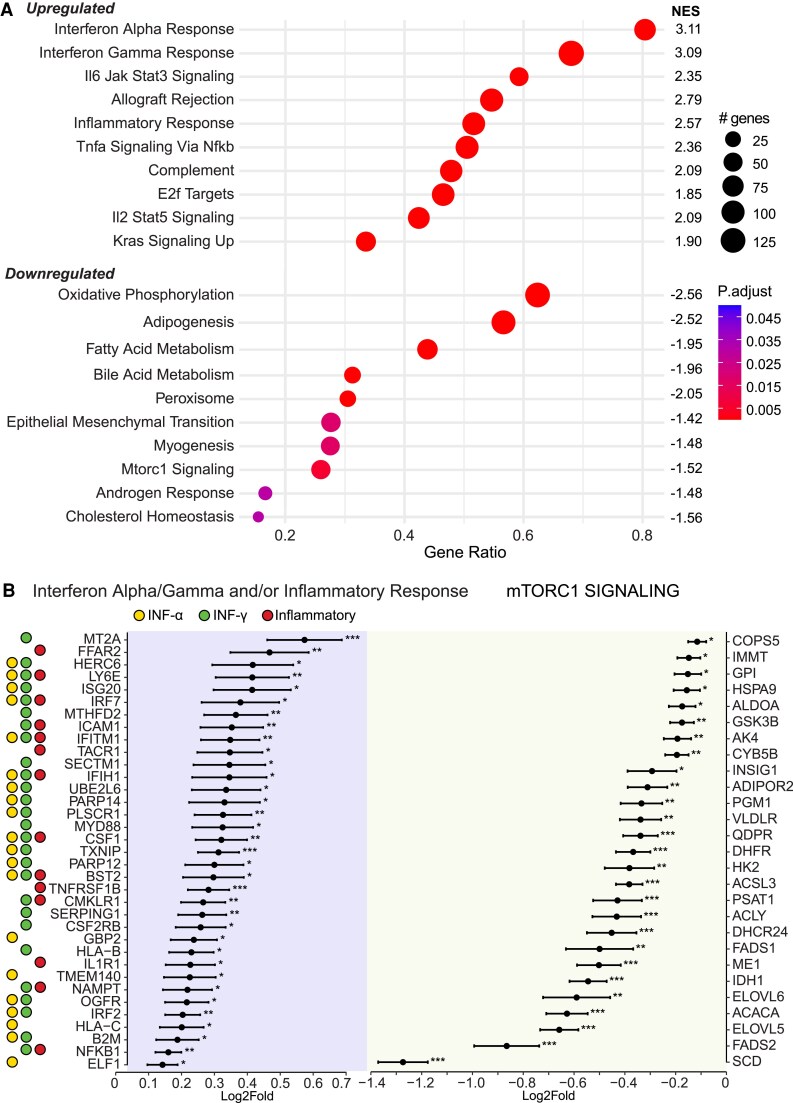
(A) Enriched Hallmark biological pathways of all genes from GSEA comparing the transcriptomics profile of AT biopsies from day 7 vs baseline. Identified genes were ranked based on log2fold value and subjected to GSEA based on predefined Hallmark gene sets (derived from MSigDB database) with a *P* value cutoff < .05 and a minimum gene set size of 25. Raw *P* values were adjusted for multiplicity by controlling the false discovery rate (FDR) with the Benjamini-Hochberg method. The dots are colored according to the adjusted *P* value and the size of the dots indicates the count of genes within each Hallmark biological pathway. The Gene Ratio refers to the ratio of input genes that were annotated in a Hallmark term. NES refers to the normalized enrichment score. (B) Top upregulated genes in the Hallmark pathways: inflammatory responses, interferon-α responses, interferon-γ responses, and mTORC1 signaling. The genes shown in the plot are those with the largest log2Fold value and a *P* < .05 in each category. The dots for each gene in the plot indicate the log2fold change, whereas the error bars represent the log2fold standard error. *P < .05, **P < .01, ***P < .001. The yellow dot indicates that the gene was identified in the interferon-α response pathway, the green dot indicates that the gene was identified in the interferon-γ response pathway, whereas the red dot indicates that the gene was identified in the inflammatory response pathway.

A total of 20 471 genes were obtained from DESeq2 analysis of the SAT RNA samples. The GSEA results (Fig. 2A) showed significant enrichments for upregulated inflammatory responses, including interferon (IFN) α and IFN-γ responses, IL-6 JAK signal transducer and activator of transcription 3 signaling, TNF-α signaling via nuclear factor kappa B (NFκB), IL-2 signal transducer and activator of transcription 5 signaling, and KRAS signaling up (FDR <0.005). Genes related to allograft rejection, complement, and E2F targets were also upregulated (FDR <0.005). Among the top upregulated genes in inflammatory responses, INF-α responses, IFN-γ responses, and TNF-α signaling via NFκB, some of them encoding receptors, proteins, or mediators involved in inflammatory responses (eg, MYD88, TNFRSF1B, CSF1, NFKB1, IL1R1) (Fig. 2B). In terms of downregulated Hallmark pathways, our analyses also showed enrichments for downregulated oxidative phosphorylation, adipogenesis, fatty acid metabolism, bile acid metabolism, peroxisome, and mechanistic Target of Rapamycin Complex 1 (mTORC1) signaling (FDR <0.005, Fig. 2A). Downregulated epithelial mesenchymal transition (FDR <0.025), myogenesis (FDR <0.025), androgen response (FDR <0.045), and cholesterol homeostasis (FDR <0.045) were also observed. Most of the top downregulated genes in mTORC1 signaling are involved in cellular energy homeostasis and fatty acid metabolism, encoding for example lipogenic and fatty acid elongation enzymes (eg, FASN, SCD, ACLY and ELOVL5, ELOVL6) and fatty acid desaturases (eg, FADS1, FADS2) (Fig. 2B). A complete overview of the GSEA leading edge genes in the top up- and downregulated Hallmark pathways is found in Table S4 (20).

### Effect of Fasting on Plasma Metabolites

**Figure 3.**
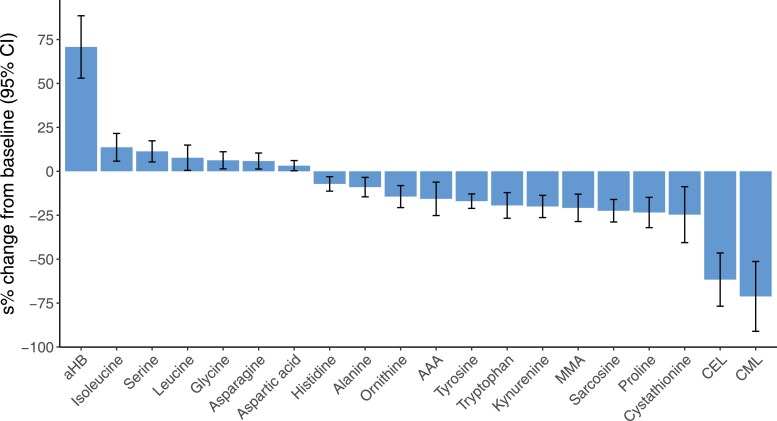
Fasting plasma metabolites showing a statistically significant change from baseline after 6 full days of fasting. Changes are shown in sympercents (95% CIs) adjusted for sex and age. Sympercents are additive and symmetric percentage differences on the log_e_ scale, calculated as the difference between the natural logs of 2 numbers multiplied by 100, ie, 100×ln(a)—100×ln(b). Abbreviations: AAA, 2-aminoadipic acid; aHB, α-hydroxybutyrate; CML, carboxymethyl-lysine; CEL, carboxyethyl-lysine; MMA, methylmalonic acid.

A total of 31 metabolites were included in the metabolite analysis (Table 2). Among these, we observed the concentrations of 20 circulating metabolites changed significantly (as defined by *P* <.05) from baseline to 7 days (Fig. 3). α-hydroxybutyrate (α-HB) showed the greatest increase, whereas the largest decrease was observed for carboxyethyl-lysine (CEL) and carboxymethyl-lysine (CML) (Table 2 and Fig. 3). As for the other plasma metabolites showing a statistically significant change, we observed an increase in isoleucine, serine, leucine, glycine, asparagine, and aspartic acid and a reduction in cystathionine, proline, sarcosine, methylmalonic acid, kynurenine, tryptophan, tyrosine, 2-aminoadipic acid, ornithine, alanine, and histidine (Table 2 and Fig. 3).

**Table 2.** Relative changes in plasma metabolites from baseline to day 7 after 6 full days of fasting*^a^*

Plasma metabolite	Baseline, gMean (1 SD ranges)	Baseline, mean (SD)	Day 7, gMean (1 SD ranges)	Day 7, mean (SD)	Relative change*^b^*	*P*-value*^c^*
α-hydroxybutyrate, µmol/L	52.4 (41.6-66.1)	53.8 (13.4)	106 (74.1-153)	113 (37.5)	103 (69.9, 142)	< .001
Isoleucine, µmol/L	76.0 (66.8-86.6)	76.6 (9.92)	87.2 (74.1-103)	88.2 (14.4)	14.6 (5.94, 24.1)	.003
Serine, µmol/L	143 (123-166)	144 (21.9)	160 (145-177)	161 (15.7)	12.0 (5.46, 18.9)	.001
Leucine, µmol/L	160 (144-177)	160 (16.1)	172 (147-202)	174 (26.1)	8.02 (0.51, 16.1)	.047
Glycine, µmol/L	267 (233-308)	270 (38.1)	285 (241-336)	288 (49.8)	6.47 (1.39, 11.8)	.020
Asparagine, µmol/L	46.7 (41.8-52.1)	46.9 (5.13)	49.5 (44.6-54.9)	49.7 (5.0)	6.02 (1.29, 11.0)	.020
Total Cysteine, µmol/L	329 (295-366)	331 (33.8)	346 (311-384)	347 (37.1)	5.05 (−0.53, 11.0)	.091
Aspartic acid, µmol/L	32.0 (28.6-35.8)	32.2 (3.47)	33.1 (29.5-37.0)	33.2 (3.59)	3.26 (0.30, 6.31)	.042
Total homocysteine, µmol/L	10.3 (8.72-12.0)	10.4 (1.66)	10.6 (8.99-12.5)	10.7 (1.68)	3.22 (−1.87, 8.57)	.232
Methionine, µmol/L	27.5 (23.8-31.6)	27.7 (3.59)	28.2 (25.6-31.2)	28.4 (2.74)	2.83 (−2.65, 8.62)	.329
Lysine, µmol/L	201 (182-222)	202 (20.1)	203 (184-224)	204 (20.4)	1.05 (−4.05, 6.42)	.697
3-hydroxyisobutyrate, µmol/L	19.6 (15.1-25.3)	20.2 (5.40)	19.7 (15.3-25.5)	20.3 (5.20)	0.84 (−8.69, 11.4)	.871
α-ketoglutarate, µmol/L	9.25 (7.26-11.8)	9.52 (2.42)	9.28 (7.30-11.8)	9.52 (2.25)	0.24 (−10.3, 12.0)	.966
Phenylalanine, µmol/L	88.7 (83.0-94.8)	88.9 (5.87)	86.8 (79.6- 94.6)	87.1 (7.68)	-2.17 (-7.23 to 3.15)	.425
Glutamine, µmol/L	554 (494-622)	557 (61.4)	540 (475-614)	544 (66.6)	-2.51 (-7.31 to 2.53)	.333
Threonine, µmol/L	144 (119-175)	147 (29.0)	138 (113-170)	141 (32.2)	-4.30 (-11.2 to 3.08)	.258
Glutamic acid, µmol/L	98.1 (74.5-129)	101 (27.0)	93.4 (71.5-122)	96.5 (26.1)	-4.78 (-11.1 to 1.97)	.175
Valine, µmol/L	303 (282-327)	304 (21.9)	287 (257-320)	288 (30.0)	-5.37 (-10.8 to 0.44)	.083
Histidine, µmol/L	80.5 (74.2-87.5)	80.8 (6.66)	75.0 (68.0-82.6)	75.3 (7.25)	-6.92 (-10.7 to -3.01)	.002
Alanine, µmol/L	417 (369-470)	420 (52.6)	381 (342-424)	383 (42.5)	-8.61 (-13.5 to -3.39)	.004
Ornithine, µmol/L	80.8 (64.1-102)	82.8 (18.6)	70.0 (60.2-81.4)	70.7 (10.8)	-13.4 (-18.7 to -7.76)	<.001
2-aminoadipic acid, µmol/L	1.24 (0.95-1.63)	1.28 (0.33)	1.06 (0.86-1.32)	1.08 (0.24)	-14.5 (-22.3 to -5.95)	.004
Tyrosine, µmol/L	79.6 (68.2-92.9)	80.4 (12.0)	67.2 (59.4-75.9)	67.6 (8.05)	-15.6 (-19.0 to -12.1)	<.001
Tryptophan, µmol/L	73.3 (63.4-84.8)	74.0 (11.3)	60.4 (50.0-72.9)	61.4 (11.9)	-17.6 (-23.5 to -11.4)	< .001
Kynurenine, µmol/L	1.92 (1.64-2.24)	1.94 (0.31)	1.57 (1.30-1.90)	1.60 (0.32)	-18.1 (-23.2 to -12.8)	< .001
Methylmalonic acid, µmol/L	0.17 (0.14-0.21)	0.17 (0.04)	0.14 (0.11-0.18)	0.14 (0.04)	-18.8 (-24.9 to -12.2)	< 0.001
Sarcosine, µmol/L	1.06 (0.87-1.28)	1.07 (0.21)	0.84 (0.71-1.00)	0.85 (0.15)	-20.1 (-25.1 to -14.8)	<.001
Proline, µmol/L	174 (135-224)	179 (48.9)	138 (122-155)	138 (17.0)	-20.9 (-27.4 to -13.8)	<.001
Cystathionine, µmol/L	0.25 (0.18-0.35)	0.26 (0.09)	0.20 (0.14-0.26)	0.20 (0.06)	-21.9 (-33.4 to -8.38)	.006
CEL, µmol/L	0.10 (0.07-0.14)	0.09 (0.04)	0.05 (0.05-0.06)	0.04 (0.01)	-46.0 (-53.6 to -37.2)	<.001
CML, µmol/L	0.09 (0.06-0.13)	0.11 (0.04)	0.04 (0.03-0.05)	0.06 (0.01)	-50.9 (-59.8 to -40.1)	<.001

Abbreviations: CEL, carboxyethyl-lysine; CML, carboxymethyl-lysine.

^*a*^Fasting plasma concentrations of the metabolites were analyzed with linear regression adjusted for age and sex. Values were transformed by the natural logarithm before the analyses.

^*b*^Relative model-adjusted mean change scores (95% CI) from baseline to follow-up as percentages calculated from the model estimates: % = (exp^estimate—^1)×100.

^*c*^ *P* values for adjusted relative changes from baseline to day 7.

^d^Unadjusted aritmethric means (SDs) and absolute change (95% CIs) of untransformed data as well as unadjusted gMean and relative changes (95% CIs) from the linear regression models are found in Tables S1-S3 (20).

#### Enriched biological pathways in gene coexpression modules associated with plasma metabolites

**Figure 4.**
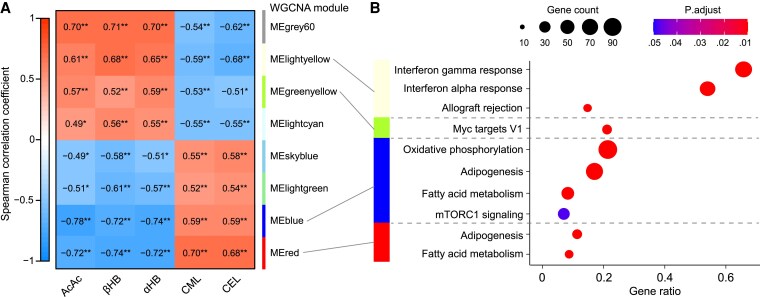
Correlation matrix of the WGCNA modules significantly correlated with selected plasma metabolites and the significantly enriched Hallmark pathways for these modules. WGCNA was performed on the gene list to identify gene coexpression network modules correlated with the most significantly altered metabolites in a module-trait analysis. An overrepresentation analysis based on predefined Hallmark gene sets (derived from MSigDB database) with a *P* value cutoff of <.05 and Benjamini-Hochberg correction was performed for modules showing a Spearman correlation coefficient of *β* > .55 or *β* < −.55 with the metabolites. (A) The scale bar on the left indicates the Spearman correlation coefficient where -1 indicates a strong negative correlation (blue color), 0 indicates no correlation, and 1 indicates a strong positive correlation (red color). (B) The dots are colored according to the adjusted *P* value and the size of the dots indicates the count of genes within each identified Hallmark biological pathway. The gene ratio refers to the ratio of input genes that were annotated in a Hallmark term. The complete correlation matrix between all identified gene coexpression network modules and metabolites can be found in Fig. S2 (20). *P < .05, **P < .01. Abbreviations: AcAc, acetoacetate; α-HB, α-hydroxybutyrate; βHB, β-hydroxybutyrate; CEL, carboxyethyl-lysine; CML, carboxymethyl-lysine.

To understand whether the most significantly altered plasma metabolites correlated with the changes in AT gene expression, WGCNA on all genes obtained from the DESeq2 analysis was performed. In total, the WGCNA analysis identified 39 gene coexpression modules that were correlated with selected metabolites (ie, plasma ketones [acetoacetate and β-HB]) and α-HB, CML, and CEL in a module-trait analysis. Figure 4A shows that these metabolites were most strongly correlated with the blue (1789 genes), red (1083 genes), lightgreen (207 genes), skyblue (101 genes), lightcyan (269 genes), greenyellow (564 genes), lightyellow (205 genes), and grey60 (242 genes) gene modules. The complete correlation matrix showing the correlation between all 39 modules and the selected plasma metabolites is found in Fig. S2 (20). The overrepresentation analysis of the separate modules (Fig. 4B) showed that the blue and red module consisted of genes involved in oxidative phosphorylation, adipogenesis, fatty acid metabolism (FDR <0.01) and mTORC1-signaling (FDR < 0.05), while the lightyellow module consisted of genes involved in IFN-α responses, IFN-γ responses and allograft rejection (FDR < 0.01). The greenyellow module consisted of genes involved in MYC-targets (FDR < 0.01). The genes in the grey60, lightcyan, skyblue, and lightgreen modules were not significantly enriched in any Hallmark pathway. Moreover, oxidative phosphorylation, adipogenesis, fatty acid metabolism, and mTORC1 signaling (blue and red modules) were inversely associated with circulating ketone (acetoacetate and β-HB) and α-HB concentrations but positively associated with CML and CEL concentrations (Fig. 4A and 4B). In contrast, IFN-α responses, IFN-γ responses, and allograft rejection (lightyellow module) and MYC targets (greenyellow module) were positively correlated with circulating ketone (acetoacetate and β-HB) and α-HB concentrations but inversely associated with CML and CEL concentrations.

#### Association between plasma metabolite changes and biochemical/anthropometric changes

**Figure 5.**
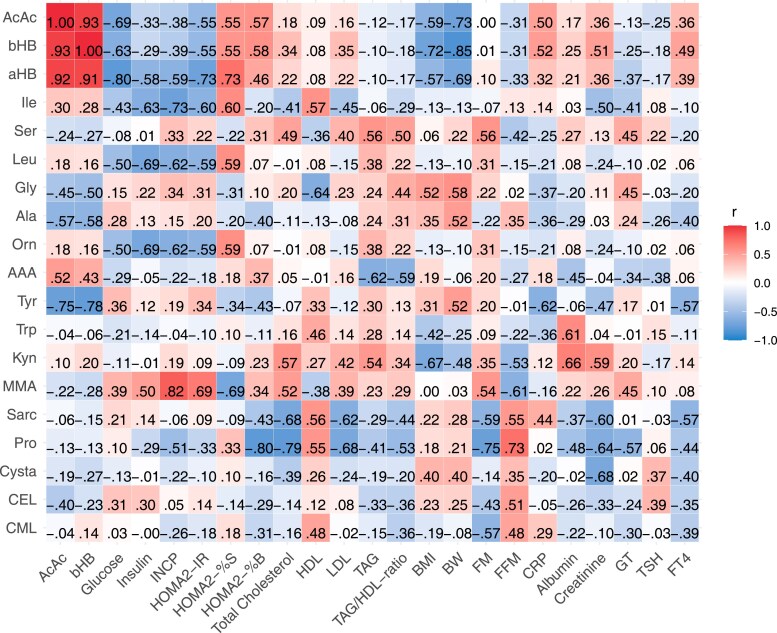
Spearman correlation matrix of the s% change in metabolites showing the largest change from baseline after 6 full days of fasting against the corresponding s% change in biochemical variables. Negative correlations are marked with a leading minus sign and colored blue while positive correlations are marked in red without a minus sign. Red color indicates positive correlations and blue color indicates negative correlations. r indicates the correlation coefficiency. The relative sympercent (s%) change in each metabolite was correlated against the relative s% change in biochemical variables. Sympercents are additive and symmetric percentage differences on the log_e_ scale, calculated as the difference between the natural logs of 2 numbers multiplied by 100, ie, 100×ln(a)—100×ln(b). Abbreviations: AAA, 2-aminoadipic acid; AcAc, acetoacetate; aHB, α-hydroxybutyrate; Ala, alanine; bHB, β-hydroxybutyrate; BMI, body mass index; BW, body weight; CEL, carboxyethyl-lysine; CML, carboxymethyl-lysine; CRP, C-reactive protein; Cysta, cystathionine; FFM, fat-free mass; FM, fat mass; FT4, free thyroxine; Gly, glycine; GT, glutamyl transferase; HDL, high-density lipoprotein; HOMA2-%B, homeostatic model assessment of β-cell function; HOMA2-%S, homeostatic model assessment of insulin sensitivity; HOMA2-IR, homeostatic model assessment for insulin resistance; Ile, isoleucine; INCP, insulin C-peptide; Kyn, kynurenine; LDL, low-density lipoprotein; Leu, leucine; MMA, methylmalonic acid; Orn, ornithine; Pro, proline; Sarc, sarcosine; Ser, serine; TAG, triacylglycerol; Trp, tryptophan; Tyr, tyrosine.

The Spearman correlation analysis of the changes in significantly altered plasma metabolites against the changes in other biochemical and anthropometrical variables is presented in Fig. 5. This analysis showed that the change in α-HB was strongly positively correlated with the change in acetoacetate, β-HB, and HOMA2-%S and negatively correlated with the change in plasma glucose and HOMA2-IR. For the change in CEL and CML, only a moderate positive correlation was observed with the change in fat-free mass. The change in isoleucine exhibited the strongest negative correlation with the change in insulin C-peptide, while the change in methylmalonic acid was most strongly positively correlated with the change in insulin C-peptide and HOMA2-IR. The change in proline showed the strongest positive correlation with the change in fat-free mass and the strongest negative correlation with the change in HOMA2-%B and total cholesterol. For tyrosine, the strongest negative correlation was observed with the change in β-HB and acetoacetate.

## Discussion

We aimed to gain new insights into the metabolic adaptation to a very-low-energy fast in humans, through a 6-day feeding study of people with obesity who donated blood and SAT before and after the intervention. A key finding is that inflammation-related gene expression in SAT increased rather than decreased, highlighting a role for inflammatory genes in the metabolic adaptation to fasting. Concomitantly, there was a marked downregulation of genes that promote adipogenesis and fatty acid β-oxidation, consistent with an overall reduced metabolic activity. These changes in SAT were correlated with increased plasma concentrations of α-HB and ketone bodies and decreased plasma concentrations of the AGEs CML and CEL.

Fasting initiates a series of metabolic adaptations aimed at maintaining energy homeostasis, including increased gluconeogenesis, glycogenolysis, fat oxidation, and ketogenesis. Notably, our study of people with severe obesity showed a greater loss of fat-free mass than loss of fat mass after a 6-day very-low-energy fast. These observed changes were similar to a previous study of 7 days fasting during exercise in healthy individuals (21), although with slightly less changes in fat mass (−1.15 vs −1.4 kg) and fat-free mass (−2.8 vs −4.6 kg) as measured by dual-energy X-ray absorptiometry. A considerable proportion of the observed fat-free mass loss can be ascribed to glycogen depletion and the associated loss of bound water, as glycogen binds 3 to 4 g of water per gram of glycogen (22), but also to some degree increased muscle proteolysis, a metabolic adaptation during fasting that supplies amino acid substrates for gluconeogenesis and ketogenesis. We observed significant changes in several glucogenic and/or ketogenic amino acids, such as increased serine, glycine, leucine, isoleucine, and decreased alanine, tryptophan, and tyrosine, as well as kynurenine, a downstream metabolite of tryptophan. We also found increased plasma concentrations of the branched-chain amino acids (BCAAs) isoleucine and leucine, which is consistent with other fasting studies (23, 24). This plasma increase in BCAA associated with muscle proteolysis might, at least in part, reflect an excess of BCAAs that is not compensated for by increased oxidation in tissues, which is further supported by accumulation of free BCAAs also within muscles initially during starvation (25). Of note, intracellular BCAAs activate the mTOR pathway during nutrient abundance as a mechanism to support cell growth (26). Although we did not measure intracellular BCAAs in muscle or adipocytes, the increased BCAA levels we observed in plasma might explain the decreased adipose expression of genes in the mTOR pathway, consistent with reduced mTOR signaling as a hallmark of the fasting response (26).

Obesity is characterized by low-grade chronic inflammation, and energy-reduced diets lower systemic and AT inflammation over time (27, 28). However, in line with our findings, a recent scoping review concluded that fasting may exert opposite effects, resulting in either no change or an increase in systemic inflammation, as indicated by elevated levels of C-reactive protein (CRP), IL-6, and TNF-α at least over shorter periods (8). These previous studies, however, were limited to changes in the circulation, and our study further reveals upregulation of genes involved in multiple inflammatory pathways in SAT, including IFN-α and IFN-γ responses, as well as TNF signaling via NFκB. Our findings further align with a 10-day zero-calorie fasting intervention in people without obesity, reporting increased inflammatory gene signatures in SAT and systemic CRP (29). In obesity, a previous study of a very-low calorie diet (800 kcal/day) in females showed no upregulation of inflammatory markers in SAT after 2 days but found that both pro- and anti-inflammatory macrophage markers were upregulated after 28 days (30). On the other hand, another study in females with obesity found no change or an increase in different inflammatory genes after a 1-month very-low calorie diet (also 800 kcal/day), but rather a reduction during weight stabilization at 3 months (31). In our study, some of the up-regulated genes are considered to typically reflect a pro-inflammatory state (eg, *MYD88*, *TNFRSF1B*, *CSF1*, *NFKB1*, *IL1R1*). However, during the adaptation to a low-energy intake, changes in these genes should not be interpreted as an overtly harmful inflammatory response but a regulatory response to facilitate a demanding metabolic transition.

Notably, during systemic inflammation, pro-inflammatory cytokines such as IFN-γ induce the enzyme indoleamine 2,3-dioxygenase, which catalyzes the conversion of tryptophan to kynurenine; this has been found to reflect reduced circulating tryptophan and increased kynurenine levels (32). The reduction we observed in plasma levels of both tryptophan and kynurenine after the 6-day fast suggests that the pro-inflammatory gene expression profile in adipose tissue was a local tissue-specific response, unlike the profile of decreased tryptophan along with increased kynurenine that is associated with systemic inflammation and mortality (32). The decrease in circulating tryptophan in our study aligns with a previous 6-day fasting study in healthy males, which also showed no increase in circulating kynurenine (although kynurenine did not decrease as in our study) (33).

Importantly, our study also shows that an increased SAT inflammatory gene expression can cooccur with improved systemic insulin sensitivity. Interestingly, mice with adipose-specific knockdown of mTORC2, mimicking a fasting cellular state and causing insulin resistance on overfeeding, was found to, in turn, cause AT inflammation (34). These data support that inflammation serves as an important response in adaptive nutrient handling by adipose tissue as a consequence of an attenuated insulin response. Our findings are furthermore consistent with a randomized controlled trial showing that a 5% weight loss neither improved systemic nor SAT inflammation despite improving insulin sensitivity (35). Although they found that a greater weight loss over time (11%-16%) lowered both inflammatory markers and insulin resistance (35), the more acute nutritional, metabolic, and hormonal state of low insulin concentrations appears to override any attenuating effect of inflammation on insulin signaling.

The increased inflammatory gene expression in SAT may be a necessary response to maintain normal AT function during the acute change in energy flux of fasting. Studies in mice have highlighted an important dynamic role for immune responses in the appropriate AT remodeling during expansion of fat mass (36), and on caloric restriction and weight loss (37). Also, pharmacological suppression of adipocyte inflammation pathways such as TNF-α impaired AT function and promoted insulin resistance in mice (38), supporting that adipose inflammation is required for maintaining normal AT function upon nutritional challenges. However, less is known about the role of inflammation-related genes in AT shrinkage in humans. Beneficial effects of inflammation-related genes in the adaptation to fasting may partly be mediated via increased autophagy, where the inflammatory mediator IFN-γ has been shown to play an important role through TNF-α-related pathways (39). Moreover, concomitant with the increased inflammation-related gene expression, we found a suppression of genes in the mTORC1 pathway, whose suppression during fasting is crucial for the induction of autophagy (40). Notably, a previous study found increased expression of 2 autophagy-related genes, GABARAPL1 and DAPK2, together with increased INF-γ signaling in human SAT in response to 26 hours of fasting (41). Our observations of increased IFN-γ and TNF signaling along with suppressed mTORC1 signaling suggest that inflammatory factors may contribute to enhanced autophagy in SAT during fasting in humans.

An important consideration is that we cannot rule out the possibility that the observed increase in inflammatory gene expression is limited to abdominal SAT. Although visceral fat tends to show a greater inflammatory potential than SAT (42), and femoral fat a lower inflammatory potential (43), further studies are needed to clarify tissue-specific effects of fasting.

Additionally, different adipose depots function differently in males and females, with females typically exhibiting a lower inflammatory potential overall (44). Given this, and the limited research on the effects of fasting on other SAT depots, it remains uncertain whether similar findings would be observed in lower body SAT and/or intraabdominal adipose depots.

An interesting finding is that plasma ketones were correlated with MYC-related processes. MYC is an oncoprotein transcription factor, influencing cell proliferation, metabolism, and other biological processes (45). Energy availability is crucial during cell proliferation, and inhibition of the nutrient sensor mTORC1 has been shown to decrease MYC translation and transcription of genes favoring cell growth (46). We would therefore have expected to observe that MYC-related genes, similarly to mTORC1, were negatively correlated with ketones. However, our data suggest that increased MYC during the acute adaptation to fasting may be involved in metabolic reprogramming to nutrient shortage. This is supported by the role of MYC as a master regulator of metabolic programming in cancer, facilitating proliferation of cancer cells despite nutrient-poor conditions by increasing alternative MYC-regulated pathways for energy such as glycolysis, glutaminolysis, and fatty acid oxidation (47). MYC can also regulate adipogenesis (48), and elevation and hyperacetylation of c-Myc in mouse adipocytes was found to promote smaller mature adipocytes with upregulated inflammatory markers, linked to loss of the obesity-protecting and antiaging NAD+ dependent enzyme deacetylase SIRT1 (49). Interestingly, although MYC can suppress autophagy (50), it can also mediate an autophagic response on cellular stress by raising reactive oxygen species production, such as during starvation (51), and by promoting unfolded protein responses and consequent antioxidant responses (52).

Our analysis of SAT also revealed downregulation of genes involved in fatty acid metabolism and the TCA cycle. This aligns with findings from the 10-day zero-calorie study by Fazeli et al, which also showed decreased expression of genes involved in lipogenesis, oxidative phosphorylation, and the TCA cycle in SAT (29). Collectively, these findings indicate an overall reduced energy turnover in SAT after a 6-day very-low-energy fast, which is also supported by the suppression of mTORC1 (26). However, the observed reduction in energy turnover in SAT not necessarily indicate reduced whole-body energy and lipid metabolism during fasting but may represent a specific energy-saving response in SAT. A study in hyperlipidemic patients with obesity on a low-calorie diet (1000 kcal/day) showed a reduction in visceral but not in subcutaneous fat mass after 14 days (53). This may suggest that visceral AT rather than SAT is the primary source of free fatty acids for lipolysis and β-oxidation in the initial phase of fasting, and visceral fat loss might have been greater than subcutaneous fat loss in our study.

Thyroid hormones are key regulators of energy metabolism, and we found that short-term fasting increased serum FT4 levels, whereas TSH levels remained unchanged. Previous studies on short- and long-term fasting have reported inconsistent findings for TSH, T4, and FT4, with some showing decreases, other increases, and some no change at all (54, 55), In contrast, more consistent patterns have been reported for serum T3, free T3 (FT3), and reverse T3, with most studies reporting reductions in T3 and FT3, alongside elevations in reverse T3 (54, 56, 57). These changes are believed to reflect a shift toward inactivated thyroid metabolism, involving both reduced peripheral conversion of T4 to the biologically active T3 and increased conversion to the biologically inactive reverse T3. Our observation of elevated FT4 could reflect this shift, representing an early adaptive shift aimed at conserving energy in response to fasting. However, because FT3 was not measured in our study, we could not fully characterize the alterations in thyroid hormone metabolism during short-term fasting.

**Figure 6.**
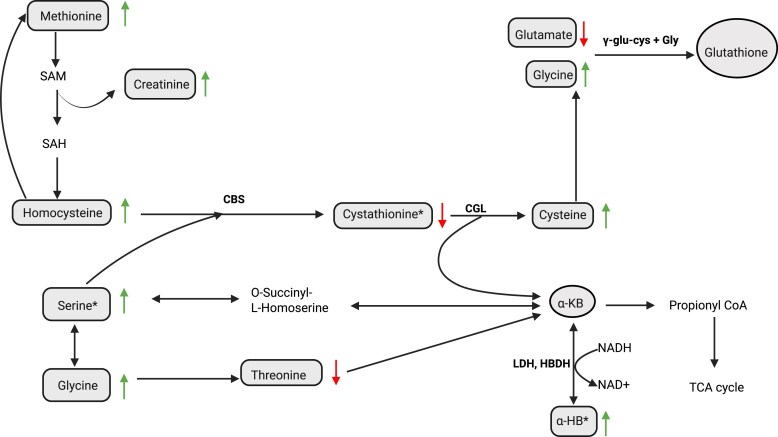
Pathways contributing to generation of α-HB. Metabolites presented in the square boxes were measured in the study, whereas those in the circular boxes were not measured. The green arrows placed beside a metabolite pointing upwards indicate an increase in circulating concentrations of this metabolite while the red arrows pointing downwards indicate a reduction in circulating concentrations of the metabolite following the very-low-energy fasting intervention. The asterisks indicate the changes that were statistically significant. Abbreviations: α-HB, α-hydroxybutyrate; α-KB, α-ketobutyrate. The only known synthesis pathway of α-HB is from α-KB via a reaction catalyzed by lactate dehydrogenase or α-hydroxybutyrate dehydrogenase. Synthesis of α-KB occurs both during the catabolism of threonine and methionine, and as part of the homocysteine transsulfuration pathway. In the latter, α-KB is produced during the conversion of cystathionine to cysteine, where cysteine subsequently can be used for glutathione synthesis. Accumulation of α-HB is believed to occur because of accumulation of α-KB when the synthesis of α-KB surpasses the rate of catabolism or due to inhibition of the enzyme converting α-KB to propionyl-CoA. Created in BioRender. Bråtveit, M. (2025) https://BioRender.com/h80d568.

During the intervention, we observed markedly increased plasma concentrations of α-HB, consistent with other fasting or very-low-calorie (800 kcal/day) studies (58, 59). Elevated α-HB, like ketone bodies, is associated with increased fatty acid oxidation, and is also linked to antioxidant production as a byproduct in the synthesis of glutathione (Fig. 6). For example, α-HB, similar to the structural glutathione analog ophthalmic acid which rises during hepatic glutathione depletion, is associated with higher glutathione requirements and impaired mitochondrial energy metabolism upon hepatic oxidative stress (60). Importantly, fasting has been shown to acutely increase hepatic oxidative stress, as indicated by elevated liver enzymes AST and ALT (61). Notably, increased α-HB has also been observed in severe ketoacidosis, which, similarly to fasting, is a condition of increased fatty acid oxidation and ketone production (62), and in mitochondrial disorders involving NADH-reductive stress (63), as well as in insulin resistance and early type 2 diabetes development (64). Importantly, we found increased plasma α-HB concentrations despite significant reductions in insulin, glucose, and HOMA2-IR. Taken together, increased α-HB appears to reflect a protective mechanism against oxidative stress by increasing glutathione production in tissues such as the liver, both in response to nutrient-rich and nutrient-poor conditions. This is further supported by the observed reduction in plasma cystathionine and increased plasma serine that feed into the synthesis of α-ketobutyrate, the only known precursor of α-HB (Fig. 6).

We also observed a great reduction in the plasma concentrations of the AGEs CML and CEL after 6 full days of fasting. AGEs are molecules generated through a nonenzymatic glycation reaction between reducing sugars such as glucose, and proteins, lipids, or nucleic acids. Accumulation of AGEs in tissues is part of the normal aging process, but excessive accumulation has been associated with several diseases (65). Biosynthesis of AGEs is associated with the degree of oxidative stress, as observed in females with type 2 diabetes (66). Similarly, glycative stress such as in hyperglycemia has been shown to exacerbate the formation of AGEs in vivo (67). Nevertheless, despite observing great reductions in serum glucose concentrations during the intervention, we found no significant correlation between changes in fasting serum glucose and plasma CML and CEL concentrations, in line with previous studies also reporting no clear relationship with glucose (68, 69). However, AGEs can also be ingested through dietary intake, with foods processed at high temperatures, such as grilled or fried foods, being particularly rich in AGEs (70). Participants in the current study were provided with a diet very low in energy with protein-based meals, likely containing much fewer dietary AGEs compared to their normal diet. The observed reduction may thus be explained by a lower dietary AGE intake. Of note, autophagy is also one of the mechanisms used by cells to avoid toxic concentrations of irreversible AGEs, as enhanced autophagy has been shown to lower AGE concentrations (71).

The current study has several strengths and limitations. First, this was a controlled in-hospital feeding study where participants were provided with all food and drinks, thus ensuring that participants followed the study diet of 400 to 600 kcal/day. However, we cannot rule out that increased stress from living in a hospital setting for a week might have, to some degree, influenced stress levels, with a possible impact on the results. Second, our study adds valuable information in humans on the role of AT, the major energy reservoir of the body, in the fasting response and its association with plasma metabolites. However, it should be recognized that other observations might have been made in visceral AT, and we were unable to evaluate simultaneous tissue-specific responses in the liver and muscle. Furthermore, we did not analyze other subcutaneous adipose depots, such as omental, gluteal, or femoral regions, which might have shown different responses than abdominal SAT. Third, although this study provides valuable knowledge about the early adaptation to fasting for both SAT and plasma metabolites, our results do not necessarily reflect long-term responses to fasting. For several of the associated health benefits of fasting, a longer intervention period may be necessary to better understand the underlying mechanisms. Fourth, because this was a limited sample of individuals with severe obesity and of Caucasian origin, the data may not necessarily be generalizable to other populations such as people without obesity. Last, the few male participants prevented us from assessing possible differences in responses between sexes.

## Conclusion

In conclusion, our study provides novel insights into the human physiological response after a 6-day very-low-energy fast in humans with obesity. In SAT, we observed a significant upregulation of inflammation-related genes, alongside a general suppression of genes involved in energy metabolism and mTORC1 signaling. The upregulation of inflammatory responses may play a role in the metabolic reprogramming required to manage altered energy flux and nutrient scarcity. Together with suppressed mTORC1 signaling, these findings may also provide indirect evidence of a fasting-induced autophagy in humans. Finally, fasting led to increased plasma concentrations of α-HB, a proposed biomarker of insulin resistance, despite notable improvements in insulin sensitivity, and a reduction in the plasma concentrations of the AGEs CML and CEL, which might reflect lowered oxidative stress.

## Data Availability

Access to the datasets is restricted (sensitive patient data) or can be made available on reasonable request pending application and approval. The transcriptomics data are publicly available at The European Genome-phenome Archive under the accession number EGAD50000001484.

## Abbreviations

α-HB: α-hydroxybutyrate
AGE: advanced glycation end-product
AT: adipose tissue
β-HB: β-hydroxybutyrate
BCAA: branched chain amino acid
CEL: carboxyethyl-lysine
CML: carboxymethyl-lysine
CRP: C-reactive protein
FDR: false discovery rate
FT3: free T3
FT4: free T4
GSEA: gene set enrichment analysis
HOMA2-%B: homeostatic model assessment of β-cell function
HOMA2-%S: homeostatic model assessment of insulin sensitivity
HOMA2-IR: homeostatic model assessment for insulin resistance
IFN: interferon
mTORC1: mechanistic Target of Rapamycin Complex 1
NFκB: nuclear factor kappa B
s%: sympercent
SAT: subcutaneous adipose tissue
TCA: tricarboxylic acid
WGCNA: weighted gene coexpression network analysis

## References

[1] Finn PF, Dice JF. Proteolytic and lipolytic responses to starvation. Nutrition. 2006;22(7-8):830‐844. 16815497 10.1016/j.nut.2006.04.008

[2] Panagiotou K, Stefanou G, Kourlaba G, Athanasopoulos D, Kassari P, Charmandari E. The effect of time-restricted eating on cardiometabolic risk factors: a systematic review and meta-analysis. Nutrients. 2024;16(21):3700. 39519533 10.3390/nu16213700 PMC11547938

[3] Longo VD, Di Tano M, Mattson MP, Guidi N. Intermittent and periodic fasting, longevity and disease. Nat Aging. 2021;1(1):47‐59. 35310455 10.1038/s43587-020-00013-3 PMC8932957

[4] Harvie M, Wright C, Pegington M, et al The effect of intermittent energy and carbohydrate restriction v. Daily energy restriction on weight loss and metabolic disease risk markers in overweight women. Br J Nutr. 2013;110(8):1534‐1547. 23591120 10.1017/S0007114513000792 PMC5857384

[5] Sutton EF, Beyl R, Early KS, Cefalu WT, Ravussin E, Peterson CM. Early time-restricted feeding improves insulin sensitivity, blood pressure, and oxidative stress even without weight loss in men with prediabetes. Cell Metab. 2018;27(6):1212‐1221.e3. 29754952 10.1016/j.cmet.2018.04.010 PMC5990470

[6] Marko DM, Conn MO, Schertzer JD. Intermittent fasting influences immunity and metabolism. Trends Endocrinol Metab. 2024;35(9):821‐833. 38719726 10.1016/j.tem.2024.04.014

[7] Pietzner M, Uluvar B, Kolnes KJ, et al Systemic proteome adaptions to 7-day complete caloric restriction in humans. Nat Metab. 2024;6(4):764‐777. 38429390 10.1038/s42255-024-01008-9 PMC7617311

[8] de Ciutiis I, Djakovic S, Cagigas ML, et al Long-term fasting and its influence on inflammatory biomarkers: a comprehensive scoping review. Ageing Res Rev. 2025;110:102797. 40484176 10.1016/j.arr.2025.102797

[9] Madeo F, Zimmermann A, Maiuri MC, Kroemer G. Essential role for autophagy in life span extension. J Clin Invest. 2015;125(1):85‐93. 25654554 10.1172/JCI73946 PMC4382258

[10] Mizushima N, Komatsu M. Autophagy: renovation of cells and tissues. Cell. 2011;147(4):728‐741. 22078875 10.1016/j.cell.2011.10.026

[11] Aman Y, Schmauck-Medina T, Hansen M, et al Autophagy in healthy aging and disease. Nat Aging. 2021;1(8):634‐650. 34901876 10.1038/s43587-021-00098-4 PMC8659158

[12] Luo L, Liu M. Adipose tissue in control of metabolism. J Endocrinol. 2016;231(3):R77‐r99. 27935822 10.1530/JOE-16-0211 PMC7928204

[13] Kersten S . The impact of fasting on adipose tissue metabolism. Biochim Biophys Acta Mol Cell Biol Lipids 2023;1868(3):159262. 36521736 10.1016/j.bbalip.2022.159262

[14] Midttun Ø, McCann A, Aarseth O, et al Combined measurement of 6 fat-soluble vitamins and 26 water-soluble functional vitamin markers and amino acids in 50 μL of Serum or plasma by high-throughput mass spectrometry. Anal Chem. 2016;88(21):10427‐10436. 27715010 10.1021/acs.analchem.6b02325

[15] Limpert E, Stahel WA. Problems with using the normal distribution–and ways to improve quality and efficiency of data analysis. PLoS One. 2011;6(7):e21403. 21779325 10.1371/journal.pone.0021403 PMC3136454

[16] Cole TJ . Sympercents: symmetric percentage differences on the 100 log(e) scale simplify the presentation of log transformed data. Stat Med. 2000;19(22):3109‐3125. 11113946 10.1002/1097-0258(20001130)19:22<3109::aid-sim558>3.0.co;2-f

[17] Love MI, Huber W, Anders S. Moderated estimation of fold change and dispersion for RNA-seq data with DESeq2. Genome Biol. 2014;15(12):550. 25516281 10.1186/s13059-014-0550-8 PMC4302049

[18] Wu T, Hu E, Xu S, et al clusterProfiler 4.0: a universal enrichment tool for interpreting omics data. The Innovation. 2021;2(3):100141. 34557778 10.1016/j.xinn.2021.100141 PMC8454663

[19] Liberzon A, Birger C, Thorvaldsdóttir H, Ghandi M, Mesirov JP, Tamayo P. The molecular signatures database (MSigDB) hallmark gene set collection. Cell Syst. 2015;1(6):417‐425. 26771021 10.1016/j.cels.2015.12.004 PMC4707969

[20] Bråtveit M, Strømland PP, Laupsa-Borge J, et al Supplementary data for “a very-low energy fast involves increased adipose inflammatory gene expression: a 6-day feeding trial (FASTOMICS-6)”. Deposited October 8 2025. doi:10.6084/m9.figshare.30304537. PMC13099190 41150634

[21] Kolnes KJ, Nilsen ETF, Brufladt S, et al Effects of seven days’ fasting on physical performance and metabolic adaptation during exercise in humans. Nat Commun. 2025;16(1):122. 39747857 10.1038/s41467-024-55418-0 PMC11695724

[22] Olsson K-E, Saltin B. Variation in total body water with muscle glycogen changes in man. Acta Physiol Scand. 1970;80(1):11‐18. 5475323 10.1111/j.1748-1716.1970.tb04764.x

[23] Schauder P, Herbertz L, Langenbeck U. Serum branched chain amino and keto acid response to fasting in humans. Metabolism. 1985;34(1):58‐61. 3965862 10.1016/0026-0495(85)90061-7

[24] Felig P, Owen OE, Wahren J, Cahill GF Jr. Amino acid metabolism during prolonged starvation. J Clin Invest. 1969;48(3):584‐594. 5773094 10.1172/JCI106017 PMC535724

[25] Holeček M . Why are branched-chain amino acids increased in starvation and diabetes? Nutrients. 2020;12(10):3087. 33050579 10.3390/nu12103087 PMC7600358

[26] Goul C, Peruzzo R, Zoncu R. The molecular basis of nutrient sensing and signalling by mTORC1 in metabolism regulation and disease. Nat Rev Mol Cell Biol. 2023;24(12):857‐875. 37612414 10.1038/s41580-023-00641-8

[27] Imayama I, Ulrich CM, Alfano CM, et al Effects of a caloric restriction weight loss diet and exercise on inflammatory biomarkers in overweight/obese postmenopausal women: a randomized controlled trial. Cancer Res. 2012;72(9):2314‐2326. 22549948 10.1158/0008-5472.CAN-11-3092 PMC3342840

[28] Moschen AR, Molnar C, Geiger S, et al Anti-inflammatory effects of excessive weight loss: potent suppression of adipose interleukin 6 and tumour necrosis factor alpha expression. Gut. 2010;59(9):1259‐1264. 20660075 10.1136/gut.2010.214577

[29] Fazeli PK, Zhang Y, O'Keefe J, et al Prolonged fasting drives a program of metabolic inflammation in human adipose tissue. Mol Metab. 2020;42:101082. 32992039 10.1016/j.molmet.2020.101082 PMC7554650

[30] Šrámková V, Rossmeislová L, Krauzová E, et al Comparison of early (2 days) and later (28 days) response of adipose tissue to very low-calorie diet in obese women. J Clin Endocrinol Metab. 2016;101(12):5021‐5029. 27715401 10.1210/jc.2016-2161

[31] Capel F, Klimcáková E, Viguerie N, et al Macrophages and adipocytes in human obesity: adipose tissue gene expression and insulin sensitivity during calorie restriction and weight stabilization. Diabetes. 2009;58(7):1558‐1567. 19401422 10.2337/db09-0033 PMC2699855

[32] Zuo H, Ueland PM, Ulvik A, et al Plasma biomarkers of inflammation, the kynurenine pathway, and risks of all-cause, cancer, and cardiovascular disease mortality: the hordaland health study. Am J Epidemiol. 2016;183(4):249‐258. 26823439 10.1093/aje/kwv242 PMC4753283

[33] Louvrou V, Solianik R, Brazaitis M, Erhardt S. Exploring the effect of prolonged fasting on kynurenine pathway metabolites and stress markers in healthy male individuals. Eur J Clin Nutr. 2024;78(8):677‐683. 38789718 10.1038/s41430-024-01451-7 PMC11300305

[34] Shimobayashi M, Albert V, Woelnerhanssen B, et al Insulin resistance causes inflammation in adipose tissue. J Clin Invest. 2018;128(4):1538‐1550. 29528335 10.1172/JCI96139 PMC5873875

[35] Magkos F, Fraterrigo G, Yoshino J, et al Effects of moderate and subsequent progressive weight loss on metabolic function and adipose tissue biology in humans with obesity. Cell Metab. 2016;23(4):591‐601. 26916363 10.1016/j.cmet.2016.02.005 PMC4833627

[36] Wernstedt Asterholm I, Tao C, Morley TS, et al Adipocyte inflammation is essential for healthy adipose tissue expansion and remodeling. Cell Metab. 2014;20(1):103‐118. 24930973 10.1016/j.cmet.2014.05.005 PMC4079756

[37] Kosteli A, Sugaru E, Haemmerle G, et al Weight loss and lipolysis promote a dynamic immune response in murine adipose tissue. J Clin Invest. 2010;120(10):3466‐3479. 20877011 10.1172/JCI42845 PMC2947229

[38] Zhu Q, An YA, Kim M, et al Suppressing adipocyte inflammation promotes insulin resistance in mice. Mol Metab. 2020;39:101010. 32408016 10.1016/j.molmet.2020.101010 PMC7272509

[39] Deretic V . Autophagy in inflammation, infection, and immunometabolism. Immunity. 2021;54(3):437‐453. 33691134 10.1016/j.immuni.2021.01.018 PMC8026106

[40] Kim YC, Guan KL. mTOR: a pharmacologic target for autophagy regulation. J Clin Invest. 2015;125(1):25‐32. 25654547 10.1172/JCI73939 PMC4382265

[41] Defour M, Michielsen CCJR, O’Donovan SD, Afman LA, Kersten S. Transcriptomic signature of fasting in human adipose tissue. Physiol Genomics. 2020;52(10):451‐467. 32866087 10.1152/physiolgenomics.00083.2020

[42] Fried SK, Bunkin DA, Greenberg AS. Omental and subcutaneous adipose tissues of obese subjects release interleukin-6: depot difference and regulation by glucocorticoid. J Clin Endocrinol Metab. 1998;83(3):847‐850. 9506738 10.1210/jcem.83.3.4660

[43] Pinnick KE, Nicholson G, Manolopoulos KN, et al Distinct developmental profile of lower-body adipose tissue defines resistance against obesity-associated metabolic complications. Diabetes. 2014;63(11):3785‐3797. 24947352 10.2337/db14-0385

[44] Goossens GH, Jocken JWE, Blaak EE. Sexual dimorphism in cardiometabolic health: the role of adipose tissue, muscle and liver. Nat Rev Endocrinol. 2021;17(1):47‐66. 33173188 10.1038/s41574-020-00431-8

[45] Jha RK, Kouzine F, Levens D. MYC function and regulation in physiological perspective. Front Cell Dev Biol. 2023;11:1268275. 37941901 10.3389/fcell.2023.1268275 PMC10627926

[46] Liu P, Ge M, Hu J, et al A functional mammalian target of rapamycin complex 1 signaling is indispensable for c-Myc-driven hepatocarcinogenesis. Hepatology. 2017;66(1):167‐181. 28370287 10.1002/hep.29183 PMC5481473

[47] Dong Y, Tu R, Liu H, Qing G. Regulation of cancer cell metabolism: oncogenic MYC in the driver's seat. Signal Transduct Target Ther. 2020;5(1):124. 32651356 10.1038/s41392-020-00235-2 PMC7351732

[48] Deisenroth C, Black MB, Pendse S, et al MYC is an early response regulator of human adipogenesis in adipose stem cells. PLoS One. 2014;9(12):e114133. 25437437 10.1371/journal.pone.0114133 PMC4250176

[49] Abdesselem H, Madani A, Hani A, et al SIRT1 limits adipocyte hyperplasia through c-Myc inhibition. J Biol Chem. 2016;291(5):2119‐2135. 26655722 10.1074/jbc.M115.675645 PMC4732199

[50] Annunziata I, van de Vlekkert D, Wolf E, et al MYC competes with MiT/TFE in regulating lysosomal biogenesis and autophagy through an epigenetic rheostat. Nat Commun. 2019;10(1):3623. 31399583 10.1038/s41467-019-11568-0 PMC6689058

[51] Toh PPC, Luo S, Menzies FM, Raskó T, Wanker EE, Rubinsztein DC. Myc inhibition impairs autophagosome formation. Hum Mol Genet. 2013;22(25):5237‐5248. 23933736 10.1093/hmg/ddt381 PMC3842180

[52] Nagy P, Varga Á, Pircs K, Hegedűs K, Juhász G. Myc-driven overgrowth requires unfolded protein response-mediated induction of autophagy and antioxidant responses in Drosophila melanogaster. PLoS Genet. 2013;9(8):e1003664. 23950728 10.1371/journal.pgen.1003664 PMC3738540

[53] Li Y, Bujo H, Takahashi K, et al Visceral fat: higher responsiveness of fat mass and gene expression to calorie restriction than subcutaneous fat. Exp Biol Med. 2003;228(10):1118‐1123. 10.1177/153537020322801004 14610249

[54] Sui X, Jiang S, Zhang H, et al The influence of extended fasting on thyroid hormone: local and differentiated regulatory mechanisms. Front Endocrinol (Lausanne). 2024;15:1443051. 39253586 10.3389/fendo.2024.1443051 PMC11381305

[55] Basolo A, Begaye B, Hollstein T, et al Effects of short-term fasting and different overfeeding diets on thyroid hormones in healthy humans. Thyroid. 2019;29(9):1209‐1219. 31298652 10.1089/thy.2019.0237 PMC6864752

[56] Boelen A, Wiersinga WM, Fliers E. Fasting-induced changes in the hypothalamus-pituitary-thyroid axis. Thyroid. 2008;18(2):123‐129. 18225975 10.1089/thy.2007.0253

[57] Merimee TJ, Fineberg ES. Starvation-induced alterations of circulating thyroid hormone concentrations in man. Metabolism. 1976;25(1):79‐83. 1246209 10.1016/0026-0495(76)90162-1

[58] Relva B, Samuelsson LM, Duarte IF, et al Changes in Serum metabolome following low-energy diet-induced weight loss in women with overweight and prediabetes: a PREVIEW-New Zealand sub-study. Metabolites. 2024;14(8):401. 39195497 10.3390/metabo14080401 PMC11356139

[59] Teruya T, Chaleckis R, Takada J, Yanagida M, Kondoh H. Diverse metabolic reactions activated during 58-hr fasting are revealed by non-targeted metabolomic analysis of human blood. Sci Rep. 2019;9(1):854. 30696848 10.1038/s41598-018-36674-9 PMC6351603

[60] Lord RS, Bralley JA. Clinical applications of urinary organic acids. Part I: detoxification markers. Altern Med Rev. 2008;13(3):205‐215. 18950247

[61] Commissati S, Cagigas ML, Masedunskas A, et al Prolonged fasting promotes systemic inflammation and platelet activation in humans: a medically supervised, water-only fasting and refeeding study. Mol Metab. 2025;96:102152. 40268190 10.1016/j.molmet.2025.102152 PMC12088818

[62] Yang W, Roth KS. Defect in α-ketobutyrate metabolism: a new inborn error. Clinica Chimica Acta. 1985;145(2):173‐182. 10.1016/0009-8981(85)90284-0 3918815

[63] Sharma R, Reinstadler B, Engelstad K, et al Circulating markers of NADH-reductive stress correlate with mitochondrial disease severity. J Clin Invest. 2021;131(2):e136055. 33463549 10.1172/JCI136055 PMC7810486

[64] Gall WE, Beebe K, Lawton KA, et al alpha-hydroxybutyrate is an early biomarker of insulin resistance and glucose intolerance in a nondiabetic population. PLoS One. 2010;5(5):e10883. 20526369 10.1371/journal.pone.0010883 PMC2878333

[65] Zhu J, Wang Z, Lv C, Li M, Wang K, Chen Z. Advanced glycation End products and health: a systematic review. Ann Biomed Eng. 2024;52(12):3145‐3156. 38705931 10.1007/s10439-024-03499-9

[66] Schindhelm RK, Alssema M, Scheffer PG, et al Fasting and postprandial glycoxidative and lipoxidative stress are increased in women with type 2 diabetes. Diabetes Care. 2007;30(7):1789‐1794. 17468356 10.2337/dc06-2585

[67] Uribarri J, del Castillo MD, de la Maza MP, et al Dietary advanced glycation end products and their role in health and disease. Adv Nutr. 2015;6(4):461‐473. 26178030 10.3945/an.115.008433 PMC4496742

[68] Miura J, Yamagishi S-i, Uchigata Y, et al Serum levels of non-carboxymethyllysine advanced glycation endproducts are correlated to severity of microvascular complications in patients with Type 1 diabetes. J Diabetes Complications. 2003;17(1):16‐21. 10.1016/s1056-8727(02)00183-6 12505751

[69] Semba RD, Arab L, Sun K, Nicklett EJ, Ferrucci L. Fat mass is inversely associated with Serum carboxymethyl-lysine, an advanced glycation End product, in Adults1,2. J Nutr. 2011;141(9):1726‐1730. 21775524 10.3945/jn.111.143172 PMC3159057

[70] Chen G, Smith JS. Determination of advanced glycation endproducts in cooked meat products. Food Chem. 2015;168:190‐195. 25172699 10.1016/j.foodchem.2014.06.081

[71] Takahashi A, Takabatake Y, Kimura T, et al Autophagy inhibits the accumulation of advanced glycation End products by promoting lysosomal biogenesis and function in the kidney proximal tubules. Diabetes. 2017;66(5):1359‐1372. 28246295 10.2337/db16-0397

